# Nuclear lamin phosphorylation: an emerging role in gene regulation and pathogenesis of laminopathies

**DOI:** 10.1080/19491034.2020.1832734

**Published:** 2020-10-25

**Authors:** Sunny Yang Liu, Kohta Ikegami

**Affiliations:** a Department of Pediatrics, The University of Chicago, Chicago, Illinois, USA; b Division of Molecular and Cardiovascular Biology, Cincinnati Children’s Hospital Medical Center, Cincinnati, Ohio, USA

**Keywords:** Nuclear lamin, lamin A/C, phosphorylation, nuclear interior, laminopathies, enhancer, chromatin, chromosome, lamina-associated domain, mitosis, c-Jun, farnesylation, interphase, cdk1, progeria, cardiomyopathies, lmna, muscular dystrophy

## Abstract

Decades of studies have established that nuclear lamin polymers form the nuclear lamina, a protein meshwork that supports the nuclear envelope structure and tethers heterochromatin to the nuclear periphery. Much less is known about unpolymerized nuclear lamins in the nuclear interior, some of which are now known to undergo specific phosphorylation. A recent finding that phosphorylated lamins bind gene enhancer regions offers a new hypothesis that lamin phosphorylation may influence transcriptional regulation in the nuclear interior. In this review, we discuss the regulation, localization, and functions of phosphorylated lamins. We summarize kinases that phosphorylate lamins in a variety of biological contexts. Our discussion extends to laminopathies, a spectrum of degenerative disorders caused by lamin gene mutations, such as cardiomyopathies and progeria. We compare the prevailing hypothesis for laminopathy pathogenesis based on lamins’ function at the nuclear lamina with an emerging hypothesis based on phosphorylated lamins’ function in the nuclear interior.

## Introduction

The nuclear lamina is a protein meshwork that covers the nuclear side of the inner nuclear membrane in animal cells. Nuclear lamins are a class of intermediate filament proteins and constitute the nuclear lamina by polymerizing and assembling into filaments. The nuclear lamina provides structural integrity to the nucleus and serves as a scaffold for interphase chromosomes by tethering heterochromatin domains to the nuclear periphery [1–3]. In addition, nuclear lamins are thought to participate in various cellular processes [4] including transcriptional regulation [5,6], chromosome organization [3], DNA damage response [7], cell signaling [8,9], cell cycle regulation [10], and mechanotransduction [11,12]. Mutations in genes encoding nuclear lamins cause a spectrum of human disorders collectively called laminopathies, including cardiomyopathies, muscular dystrophies, and the premature aging disorder Hutchinson-Gilford progeria [13]. The molecular mechanisms by which nuclear lamins participate in various biological processes remain elusive, as do the pathogenic mechanisms underlying laminopathies.

There are two nuclear lamin types, A-type and B-type [14]. A-type lamins include Lamin A and Lamin C (Lamin A/C), two splice isoforms encoded by *LMNA* in humans. B-type lamins include Lamin B1 encoded by *LMNB1* and Lamin B2 encoded by *LMNB2* in humans. The A-type lamin gene arose in vertebrate evolution from the ancestral B-type lamin genes, which are conserved across metazoans [15]. Each lamin subtype forms separate lamin polymers and filaments in the nuclear lamina [6,16]. A-type lamins are expressed robustly in differentiated cells but nearly undetectable in pluripotent stem cells and during early embryogenesis [17–20]. In contrast, B-type lamins are thought to be expressed in every cell [21,22]. The biological significance of the cell-type specificity and species specificity of different lamin subtypes is poorly understood.

Nuclear lamins have also been observed in the interior of the nucleus of interphase cells [6,23–25]. Nuclear-interior lamins were originally thought to constitute a macromolecular ‘nuclear matrix’, a hypothetical chromatin scaffold in the nuclear interior [24,26]. However, recent studies have found that at least some fraction of nuclear-interior lamins are soluble, mobile, and unpolymerized [6,27–30]. Thus, nuclear-interior lamins exhibit molecular features starkly different from those of polymer lamins at the nuclear lamina.

Phosphorylation of nuclear-peripheral lamins provides the mechanistic basis for nuclear lamina disassembly during the mitosis phase of the cell cycle. Nuclear lamin phosphorylation causes lamin depolymerization at the onset of mitosis for nuclear envelope breakdown [31–33]. At the end of mitosis, nuclear lamins are dephosphorylated and reassembled into polymers in the nuclear lamina. Lamin phosphorylation has also been observed in interphase [34,35], but the molecular details of interphase-phosphorylated lamins had been obscure until recently. Recent studies found that interphase phosphorylation marks a fraction of nuclear lamins in the nuclear interior [27–29]. Furthermore, some phosphorylated lamins in the nuclear interior bind to genomic regions characteristic of gene enhancers in the human genome [28]. Thus, a focus on phosphorylation of nuclear lamins has opened a new avenue for investigating nuclear lamin functions in the cell.

In this review, we summarize the current understanding of molecular features, localization, regulation, and functions of phosphorylated nuclear lamins. We distinguish the various cellular pathways through which lamins are phosphorylated. We discuss our recent observation suggesting that phosphorylated lamins act as transcriptional activators at enhancers in the nuclear interior. Finally, we extend our discussion to the ways in which laminopathy-causing mutations might influence lamin phosphorylation and the functions of phosphorylated lamins, offering new hypotheses for the pathogenesis of laminopathies.

## Lamin phosphorylation and nuclear lamina disassembly during mitosis

**Figure 1.**
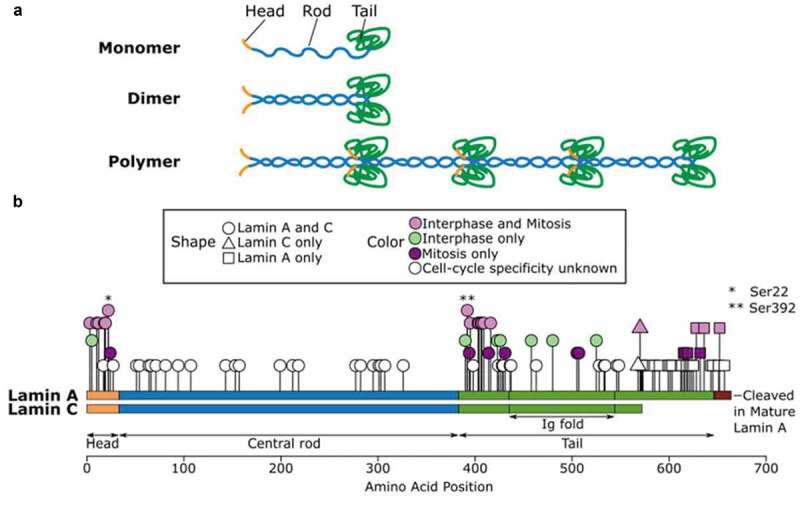
Lamin polymerization and phosphorylation. (a) Schematic representation of lamin polymerization. Lamins form dimers through rod domain interactions. Lamin dimers associate longitudinally into polar head-to-tail polymers. (b) Distribution of phosphorylation sites in Lamin A/C. Phosphorylation sites are stratified by the cell-cycle phase in which the residue is reported to be phosphorylated

Nuclear lamins are composed of three structural domains: the short N-terminal head domain (aa1-33 in human Lamin A/C; amino acid position in UniProtKB P02545), the central rod domain (aa34-383 in Lamin A/C), and the C-terminal tail domain (aa384-646 in Lamin A) [36–40] Figure 1a, B. The tail domain includes an immunoglobulin (Ig) fold domain (aa436-544) that harbors various protein and DNA interacting sites [41]. The central rod domains of two lamin molecules interact in parallel to form dimers [38]. Lamin dimers then interact in a head-to-tail fashion to form polymers, with the tail domain being protruded out of the polymer axis [38,42,43]. Lamin polymers further interact in an antiparallel fashion to form tetrameric filaments [44].

Phosphorylation of nuclear lamins reaches the highest level at the onset of the mitosis phase of the cell cycle to disassemble the lamin polymers [42,43,45]. Mitotic lamin phosphorylation predominantly occurs at two residues flanking either side of the central rod domain, often called ‘mitotic sites’, which are Ser22 and Ser392 in Lamin A/C Figure 1b, Ser23 and Ser393 in Lamin B1 (amino acid position in UniProtKB P20700), and Thr34, Ser37, and/or Ser405 in Lamin B2 (amino acid position in UniProtKB Q03252) [31,32]. Consistent with the presence of these pairs of mitotic sites, Lamin A, Lamin B, Lamin C have approximately 2 moles of associated phosphate per mole of lamin during mitosis [46]. Evidence suggests that every Lamin A/C molecule is phosphorylated at Ser22 during mitosis [28]. Phosphorylation at the two mitotic sites induces lamin depolymerization *in vitro* [33,47] and is required for nuclear lamina disassembly *in vivo* [48]. Evidence also suggests that depolymerized lamins are dimers during mitosis [16]. Conversely, dephosphorylation of the mitotic sites is required for nuclear lamin polymerization *in vitro* [47] and nuclear lamina assembly *in vivo* [49]. In addition to the two canonical mitotic sites, 28 other serine and threonine residues in Lamin A/C have been reported to exhibit increased phosphorylation during mitosis [27,35,50] (Supplementary Table 1). Many of these residues also flank the rod domain Figure 1b, although the contribution of these additional phosphorylations to lamin depolymerization during mitosis is not well understood.

There are several interesting differences between A-type and B-type lamins in their localization during mitosis. Lamin A/C are dissociated from the nuclear membrane and dispersed throughout the mitotic cytoplasm upon phosphorylation and depolymerization. In contrast, B-type lamins remain associated with the remnants of the nuclear membrane [46,51]. The association of B-type lamins with the remnants of the nuclear membrane is thought to be mediated by their C-terminal farnesylation, which is absent in Lamin A/C. Lack of farnesylation in Lamin A is due to the protease-mediated cleavage of the C-terminus during Lamin A maturation, and this cleavage site is encoded in an exon acquired during *LMNA* gene evolution in vertebrates [15]. Lamin C lacks the farnesylation site altogether. Toward the end of mitosis, both A-type and B-type lamins accumulate on the surface of condensed telophase chromatin, but in different ways [52,53]. Lamin A/C accumulation starts at the central region of telophase chromatin (called the ‘core’ region) and this process depends on the prior localization of Lamin A/C-interacting protein BAF (Barrier-to-Autointegration Factor) at the core region [52,53]. In contrast, Lamin B1 accumulation does not begin at the core and the process is independent of BAF [52,53]. Lamin A/C remain phosphorylated when localized to telophase chromatin [54], and evidence suggests that Lamin A/C are dephosphorylated on the telophase chromatin surface for repolymerization [55]. Whether B-type lamins are also dephosphorylated on the telophase chromatin surface has not been explored. These differences of mitotic localization between A-type and B-type lamins might be related to the observation that B-type lamins promote assembly of the mitotic spindles during mitosis, while A-type lamins appear to lack this function [56]. Whether A-type lamins have specific functions during mitosis is not known.

## Nuclear lamin phosphorylation in interphase

The first report that nuclear lamins are phosphorylated in interphase dates to 1980 [34], although the biological significance of interphase phosphorylation had long been obscure until recently. One study estimated that the level of interphase lamin phosphorylation is 4–7 times lower than their mitotic phosphorylation level (therefore 0.3–0.5 moles of phosphates per mole of lamin) [46], suggesting that only a subset of lamins undergo phosphorylation during interphase. Compared to Lamin A/C, interphase phosphorylation of B-type lamins has been much less investigated [57,58]. Reviewing the literature, we identified 92 total phosphorylation sites reported for Lamin A and/or Lamin C in any cell cycle stage [27,35,50,59–64] (Figure 1b; Supplementary Table 1). Of the 92 phosphorylation sites in Lamin A/C, 25 are known to be phosphorylated during interphase in human HeLa or murine A9 cell lines [27,35]. Eighteen of the 25 interphase phosphorylation sites in Lamin A/C are also reported to be phosphorylated during mitosis, including Ser22 and Ser392, the canonical mitotic sites. In fibroblasts, Ser22-phosphorylated Lamin A/C is observed in G1, S, and G2 phases of interphase, with some variability in the Ser22 phosphorylation level between interphase cells [28]. Consistent with the notion that Ser22 phosphorylation drives lamin depolymerization, Ser22-phosphorylated Lamin A/C in interphase are localized in the nuclear interior, not at the nuclear periphery [27,28]. Lamin A with phospho-mimetic Ser22Asp or phospho-mimetic Ser392Asp substitutions are highly mobile in interphase nuclei [27], suggesting that Ser22 and Ser392-phosphorylated Lamin A/C in interphase represent unpolymerized Lamin A/C. Unlike during mitosis, however, the nuclear lamina appears intact in interphase cells with Ser22-phosphorylated Lamin A/C present in the nuclear interior [28]. Furthermore, the Ser22-phosphorylated population appears to represent a small fraction of all Lamin A/C molecules in the interphase nucleus [28]. Thus, interphase Ser22-phosphorylation occurs in a small fraction of Lamin A/C and does not induce depolymerization of the entire nuclear lamina in normal cells. Phospho-mimetic substitution of Lamin A/C at Ser390, Ser404, or Ser407 also promotes relocalization of Lamin A/C to the nuclear interior, similarly to Ser392 and Ser22 phosphorylation [27]. Evidence suggests that Ser403 phosphorylation promotes nuclear import of Lamin A/C, while Ser628 phosphorylation restricts nuclear import [27,65]. Given the large overlap between interphase and mitotic phosphorylation sites, some phosphorylated nuclear lamins in interphase might be carryovers from lamin phosphorylation in mitosis. Alternatively, nuclear lamins may be phosphorylated *de novo* in interphase by kinases active in interphase.

We recently observed that Lamin C is more strongly phosphorylated at Ser22 than Lamin A in interphase fibroblasts. This high degree of Lamin C phosphorylation may be related to the previous observation that Lamin C is more soluble than Lamin A in interphase cells [16]. What makes Lamin C more susceptible to phosphorylation than Lamin A? Unlike Lamin A and B-type lamins, Lamin C does not undergo farnesylation, and consequently, newly synthesized Lamin C is thought to populate the nucleoplasm first before becoming incorporated into the lamina. On the other hand, newly synthesized precursor Lamin A and B-type lamins are directly incorporated into the nuclear lamina through the contiguous ER membrane/nuclear membrane structure to which they are tethered by farnesylation [66]. A prediction based on this difference in the lamina incorporation pathways is that Lamin A and B-type Lamins build the foundation of the nuclear lamina meshwork, on which Lamin C meshwork assembles. While exact localization of Lamin C within the nuclear lamina has not yet been defined, a recent study using super-resolution microscopy reported that localization of Lamin A/C (detected by an antibody recognizing both Lamin A and C) is closer to the nuclear interior than Lamin B1 within the nuclear lamina meshwork [67]. Thus, one possibility is that Lamin C is most accessible by kinases of all lamin subtypes within the nuclear lamina by virtue of their differential localization within the nuclear lamina.

In summary, the nuclear-interior localization of phosphorylated forms of lamins presents the exciting possibility that phosphorylated lamins may have unexplored functions distinct from nuclear-peripheral polymerized lamins. In the next section, we discuss regulators of lamin phosphorylation.

## Kinases and phosphatases for nuclear lamins

**Figure 2.**
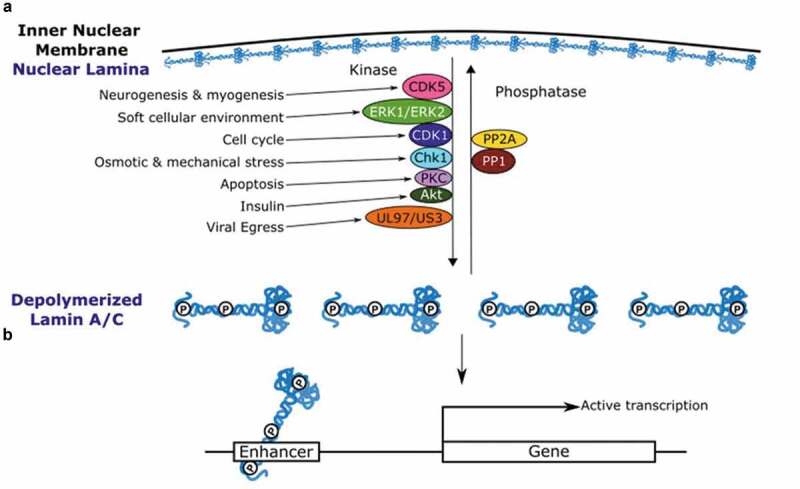
Regulators and functions of lamin phosphorylation. (a) Kinases and phosphatases known to regulate lamin phosphorylation are shown. Phosphorylation of lamins could cause depolymerization and localization to the nuclear interior. (b) Phosphorylated Lamin A/C bind to enhancers of active genes in the nuclear interior

Numerous kinases and phosphatases for nuclear lamins have been identified in a variety of biological contexts (Figure 2; Supplementary Table 1). Cyclin-Dependent Kinase 1 (CDK1) and Protein Kinase C (PKC) phosphorylate nuclear lamins at the onset of mitosis. CDK1 becomes active specifically at the onset of mitosis after forming a complex with Cyclin B. The CDK1-Cyclin B complex phosphorylates Thr19, Ser22, and Ser392 of Lamin A/C, Ser23, and Ser393 of Lamin B1, and Thr34, Ser37, and Ser405 of Lamin B2 for lamin depolymerization in mitosis [31,57,68,69]. PKC phosphorylates Ser395 and Ser405 of Lamin B1 during mitosis [70], and evidence suggests that PKC also phosphorylates Ser5, Ser395, Thr416, and Ser572 of Lamin A/C [35]. While neither the CDK1-Cyclin B1 complex nor PKC is known to accumulate specifically at the nuclear envelope at the onset of mitosis [71–73], there is little doubt that lamin phosphorylation predominantly takes place at the nuclear periphery because phosphorylation is required for depolymerization of nuclear-peripheral lamins [31–33]. The current model suggests that phosphorylation of nuclear lamins as well as other nuclear envelope proteins by CDK1, PKC, and other kinases culminates in nuclear envelope breakdown in mitosis [66]. Unlike CDK1 [74], PKC activity itself is not restricted to mitosis [75]. Nuclear lamins are dephosphorylated at the end of mitosis (telophase) for nuclear lamina reformation, and this process is mediated by phosphatases. Phosphatases PP1 and PP2A dephosphorylate Lamin A/C at Thr19 and Ser22 [76] and Lamin B2 [77] and are required for nuclear envelope reassembly upon mitotic exit [78,79]. PP1 is known to accumulate on the surface of telophase chromatin, as are lamins [55,79]. Therefore, lamins are likely dephosphorylated on the chromatin surface for repolymerization. The activity of PP1/PP2A and CDK1 is mutually antagonistic [80], providing the mechanistic basis for the phosphorylation–dephosphorylation cycle of nuclear lamins during mitosis. Beyond the mitotic exit, PP1 and PP2A become active in various other biological contexts such as glycogen [81] and sphingolipid metabolism [82], as well as the DNA damage response [83], potentially contributing to regulation of lamin phosphorylation in non-mitotic contexts.

Among lamin kinases active in interphase are ERK1 and ERK2 (also known as MAP kinases), two closely related kinases active under various extracellular stimuli. ERK1 and ERK2 phosphorylate Lamin A/C at Ser22 [84]. Ser22 of Lamin A/C is within both the CDK and ERK recognition sequence motifs. ERK1 and ERK2 can directly interact with Lamin A and Lamin C *in vitro* and *in vivo* [85]. An interesting possibility is that ERK1 and ERK2 phosphorylate Lamin A/C for nuclear-interior localization in interphase in response to various conditions triggering cellular stress. One such condition may be the type of soft extracellular environment in which Ser22 and Ser392 phosphorylation is known to be promoted [29,86]. In this context, nuclear-interior localization of phosphorylated Lamin A/C is thought to facilitate rounding of the nucleus in response to rounding of the cell under soft microenvironments [29,86]. Another lamin kinase active in interphase is Akt (also known as Protein Kinase B), active in many cellular processes including glucose metabolism [87]. Akt phosphorylates Lamin A/C at Ser404 in the mouse myoblast C2C12 cell line, and this phosphorylation is promoted by insulin [88,89]. Akt also phosphorylates Ser458 of Lamin A/C in muscle tissues isolated from *LMNA*-related myopathy patients [90]. In addition, a proteome study identified Thr10, Ser406, Ser407, Thr409, Ser414, Thr416, and Thr548 of Lamin A/C as substrates of Akt and ribosomal S6 kinases in cancer cell lines [62].

Several lamin kinases are known to promote extensive modulation of the nuclear lamina in interphase cells. PKC-δ phosphorylates B-type lamins for nuclear lamina disassembly during apoptosis [91,92]. During sea urchin fertilization, PKC in oocytes phosphorylates Lamin B1 of the sperm nuclei, leading to dissolution of the sperm nuclear lamina required for male pronucleus formation [93]. CDK5, a member of the cyclin-dependent kinase family that does not require cyclins for activation [94], phosphorylates Ser22 and Ser392 of Lamin A/C and Ser23 and Ser393 of Lamin B1 in the mouse neuronal HT22 cell line [95]. Evidence suggests that an aberrant increase of CDK5 activity in neuronal cells causes nuclear dispersion through lamin disassembly, resulting in neuronal death in Alzheimer’s disease [95]. CDK5 activity has also been reported in non-neuronal cells, such as in muscle, in which CDK5 promotes differentiation of myoblasts [94], although the relationship between lamin phosphorylation and myogenesis is unclear. Chk1, a kinase that coordinates cell-cycle arrest with DNA damage response, phosphorylates Ser307 of Lamin A/C [96]. Chk1 is known to localize at the nuclear periphery upon mechanical and osmotic stress and is thought to contribute to structural alteration of the nuclear envelope [97]. Therefore, Ser307 phosphorylation of Lamin A/C may contribute to the structural change of the nuclear lamina upon mechanical or osmotic stress.

Finally, nuclear lamin phosphorylation can be catalyzed by viral kinases for virus egress. UL97, a CDK1-like kinase of human cytomegalovirus (HCMV), phosphorylates Lamin A/C at Ser22 [98]. Consistently, HCMV infection dramatically increases Ser22-phosphorylated Lamin A/C levels in the nuclear interior [99]. UL97-mediated Ser22 phosphorylation is recognized by prolyl isomerase PIN1 to promote Lamin A/C depolymerization [99]. The US3 kinase of herpes simplex virus type 1 (HSV-1) also phosphorylates Lamin A/C [100], and a study suggests the target residues are Ser22 and Ser392 [99].

In summary, a number of kinases and phosphatases operate on nuclear lamins within diverse biological contexts, sometimes at identical residues. It is plausible that these kinases and phosphatases regulate the equilibrium between polymer lamins and unpolymerized lamins in the interphase nuclei (Figure 2a). What remain to be defined are the subcellular locations at which lamin phosphorylation and dephosphorylation take place during interphase. Furthermore, the functional significance of lamin phosphorylation is poorly understood apart from structural modulation of the nuclear lamina. In the next section, we discuss a new direction of research suggesting that phosphorylated lamins have specific functions in gene regulation in the nuclear interior.

## Phosphorylated lamin c at enhancers

Nuclear lamins exhibit a high affinity to DNA and chromatin, with a nano-molar range dissociation constant (K_D_) for interactions between the C-terminal domain of Lamin A/C and DNA or nucleosomes [101]. Polymer nuclear lamins at the nuclear periphery interact with large heterochromatin domains called lamina-associated domains (LADs), which contain mostly transcriptionally inactive genes [102–106]. By tethering LADs to the nuclear periphery, nuclear lamins influence the spatial organization of chromosomal regions [1]. Evidence suggests that nuclear lamins also promote transcriptional repression of some of the genes embedded in LADs [107–109]. Given the chromatin binding property of nuclear lamins, one hypothesis had been that phosphorylated lamins in the nuclear interior might also bind chromatin, but at different locations than LADs.

We recently investigated the genomic distribution of Ser22-phosphorylated Lamin A/C in interphase human fibroblast cells [28]. Using an antibody specific to Ser22 phosphorylation of Lamin A/C in chromatin immunoprecipitation coupled with high-throughput sequencing (ChIP-seq), we observed that Ser22-phosphorylated Lamin A/C interacts with numerous genomic sites with features of active enhancers, near genes undergoing active transcription. The enhancer-like features of Ser22-phosphorylated Lamin A/C-binding sites are in stark contrast to transcriptionally-inactive, megabase-wide heterochromatin features of LADs [1,102–106]. As described earlier, Ser22-phosphorylated Lamin C is more abundant than Ser22-phosphorylated Lamin A in interphase fibroblasts. Consistent with this observation, Lamin C with phospho-mimetic Ser22Asp and Ser392Asp substitutions binds more strongly to putative enhancers than Lamin A with the same phospho-mimetic substitutions [28]. These observations suggested that Lamin C is the primary form binding to putative enhancers upon phosphorylation. Thus, phosphorylated Lamin C may act as a transcriptional activator directly regulating transcription at enhancers in the nuclear interior Figure 2b.

In what biological context might the enhancer binding of Ser22-phosphorylated Lamin C be promoted? Our study revealed that Ser22-phosphorylated Lamin C-bound sites overlap almost exclusively with the genomic sites occupied by the AP-1 transcription factor c-Jun [28]. c-Jun is activated by multiple kinases including JNKs (Jun-N-terminal kinases) and ERK upon various extracellular stimuli [110]. While direct interaction between Lamin A/C and c-Jun has not been reported, Lamin A/C is known to interact with c-Fos, the binding partner of c-Jun in the AP-1 transcription factor complex, at the nuclear periphery [85,111]. Evidence suggests that c-Fos is unphosphorylated and inactive when interacting with Lamin A/C at the nuclear periphery, and ERK2-dependent phosphorylation of c-Fos at the nuclear lamina relocalizes c-Fos to the nuclear interior for DNA binding [85]. One hypothesis based on these observations is that Lamin C and c-Jun/c-Fos might be phosphorylated together at the nuclear periphery by ERK2 and directed to AP1-target enhancers. Overall, an attractive model is that various cellular conditions that promote lamin phosphorylation result in a transcriptional response directly mediated by phosphorylated Lamin C binding to gene enhancers Figure 2.

### The lamin A/C-LAP2α complex

Several studies have utilized chromatin fractionation to probe chromatin regions associated with nuclear-interior lamins [112–114]. Lund et al. performed ChIP-seq using an antibody that detects total Lamin A/C but in a soluble fraction obtained by micrococcal nuclease digestion, a mild lysis condition that enriches unpolymerized lamins [112]. This procedure identified low-level continuous enrichment of Lamin A/C across large regions outside of LADs [112]. These Lamin A/C-associated regions were over-represented for histone modifications associated with transcriptional repression [112]. A similar observation was made via total Lamin A/C ChIP-seq in a soluble fraction obtained by mild mechanical DNA shearing [113]. Lamin A/C-associated regions found via this procedure overlapped genomic regions bound by LAP2α, a Lamin A/C-binding protein exclusively localized in the nuclear interior [113,115,116]. Therefore, LAP2α-associated nuclear-interior Lamin A/C likely bind to transcriptionally inactive regions outside of LADs. The difference in genomic localization profiles between LAP2α-associated Lamin A/C and Ser22-phosphorylated Lamin C (which binds to putative active enhancers) suggests that LAP2α-associated Lamin A/C and Ser22-phosphorylated Lamin C represent distinct pools of nuclear-interior Lamin A/C. The interaction between Lamin A/C and LAP2α is mediated by the C-terminal region of Lamin A/C (amino acids 319–572), which harbors numerous interphase phosphorylation sites [116]. One possibility is that phosphorylation and dephosphorylation within the C-terminal region affects the Lamin A/C–LAP2α interaction, thereby regulating an exchange between LAP2α-associated and non-associated Lamin A/C in the nuclear interior.

The function of the LAP2α has been investigated extensively [117,118]. LAP2α binds Lamin A/C and is thought to retain Lamin A/C in the nuclear interior during the G1 cell-cycle phase [119,120]. Lamin A/C and LAP2α interact with Retinoblastoma Protein (RB) [121,122], a repressor of the E2F-mediated G1-to-S cell cycle transition [123]. Lamin A/C deletion results in reduction of RB abundance, presumably due to an increased susceptibility of RB to proteasome-mediated degradation [124,125], and promotes cell-cycle progression into the S-phase [124]. Similarly, LAP2α-knockout cells are defective in cell cycle arrest [119]. These studies suggest that LAP2α-associated nuclear-interior Lamin A/C protects RB from degradation, thereby negatively regulating cell proliferation. Whether Ser22-phosphorylated Lamin A/C participates in regulation of RB has not been explored.

## Laminopathies

There are over 200 known autosomal-dominant point mutations in *LMNA* that cause human disease. These diseases, collectively called laminopathies, include cardiomyopathies, muscular dystrophies, lipodystrophies, peripheral neuropathies, and Hutchinson-Gilford progeria [126]. Pathogenic mutations in *LMNB1* and *LMNB2* are much less frequent [127], possibly due to the perinatal requirement of *LMNB1* and *LMNB2* as opposed to postnatal requirement of *LMNA* [128–130]. The laminopathy mutations in *LMNA* cause nonsynonymous substitutions in the vast majority of cases. There is no apparent relationship between the amino acid locations of the mutations and disease phenotypes except in a few cases [14]. There is a phenotypic overlap among certain laminopathies such as Emery-Dreifuss muscular dystrophy type 2 (EDMD2) and dilated cardiomyopathy type 1A (CMD1A), both of which affect the cardiac muscle [131]. Although the mechanisms by which *LMNA* mutations cause laminopathies remain unknown, a number of molecular changes have been reported in cells or tissues derived from laminopathy patients or in animal or cellular models of laminopathies. These molecular changes include abnormal gene expression [132], abnormal cell signaling [133], increased DNA damage [134], abnormal localization of telomeres [135], altered nuclear shape [136], and altered response to mechanical stress [137]. A challenge in finding treatment for laminopathies has been to identify the molecular changes that trigger their pathogenesis and distinguish these upstream molecular changes from downstream molecular changes.

## The prevailing hypotheses for laminopathies

There have been two prevailing, non-mutually exclusive hypotheses for the pathogenic mechanism underlying laminopathies, both based on lamins’ functions at the nuclear periphery [13]. The gene expression hypothesis states that laminopathy mutations disrupt interactions between the nuclear lamina and LADs, leading to abnormal gene expression. The structural hypothesis states that laminopathy mutations render the nuclear envelope structurally defective, causing dysregulation of intranuclear processes.

The gene expression hypothesis has been studied extensively in the context of Hutchinson-Gilford progeria syndrome (HGPS), a premature aging syndrome caused by heterozygous *LMNA* mutations [138]. HGPS is caused predominantly by a mutation within the Lamin A-specific region of the *LMNA* gene that activates cryptic splicing and results in a mutant Lamin A protein called ‘progerin’ [138]. Progerin lacks the C-terminal cleavage site that is used to detach the farnesylated C-terminal end in normal Lamin A processing, thus being permanently farnesylated [138]. Progerin accumulates at the nuclear periphery due to this permanent farnesylation, and progerin accumulation has been hypothesized to cause disorganization of heterochromatin at lamina-associated domains (LADs) [139]. Supporting this hypothesis, in progeria-patient fibroblasts, some LADs lose interactions with nuclear-peripheral Lamin A, and the losses of LADs coincide with losses of histone H3 trimethylation at lysine 9 and lysine 27, two modifications that mark heterochromatin [28,140]. Similar losses of heterochromatin have been observed by immunofluorescence and electron microscopy in progeria-patient cells [140,141].

Recently, several groups, including ours, have conducted detailed analyses to define whether losses of heterochromatin are responsible for dysregulated gene expression in laminopathies. Our parallel analysis of gene expression, lamina–chromatin interaction, and histone modifications revealed that only a very small number of dysregulated genes are located within lost LADs in progeria-patient fibroblasts, although LAD losses do accompany losses of heterochromatin-associated histone modifications [28]. Similar observations have been reported for other laminopathies. Lee et al. found increased chromatin accessibility within LADs, indicative of losses of heterochromatin, in the cardiomyocytes differentiated from the induced pluripotent stem cells (iPSC) derived from *LMNA*-related dilated cardiomyopathy patients [142]. However, increased chromatin accessibility within LADs was not directly responsible for abnormal activation of platelet-derived growth factor (PDGF) signaling that caused arrhythmic phenotypes of the mutant cardiomyocytes [142]. Bertero et al. performed chromatin conformation analysis in cardiomyocytes differentiated from other *LMNA*-related cardiomyopathy-patient iPSCs and identified genomic regions that lose heterochromatin [143]. Again, the heterochromatin change did not explain most gene expression alterations in the mutant cardiomyocytes [143]. Together, these independent studies suggest that the alteration of LADs is unlikely to be a major contributor to abnormal gene expression changes in laminopathies.

## Phosphorylated lamin C–enhancer binding is altered in progeria

An emerging new hypothesis, drawing upon evidence provided by recent reports on phosphorylated lamin activity, is that impaired functions of phosphorylated Lamin A/C in the nuclear interior underlie the pathogenesis of laminopathies. We therefore recently examined whether interactions between Ser22-phosphorylated Lamin C and enhancers are altered in the fibroblasts derived from progeria patients. This investigation led us to observe that a specific subset of enhancer-like elements either gain or lose interactions with Ser22-phosphorylated Lamin C in progeria [28]. Consistent with the hypothesis that Ser22-phosphorylated Lamin C acts as a transcriptional activator, gains of Ser22-phosphorylated Lamin C binding were correlated with acquisition of histone acetylation and c-Jun binding at the binding sites, and losses with reduction of histone acetylation and c-Jun binding in progeria-patient cells [28]. Furthermore, gains and losses of Ser22-phosphorylated Lamin C binding were accompanied by increased and decreased expression of nearby genes in progeria-patient cells, respectively. In particular, we found that abnormally activated genes with nearby gains of Ser22-phosphorylated Lamin C are important in the pathophysiology of progeria [28]. In these progeria-patient cells, progerin itself was not phosphorylated at Ser22, and the phosphorylation level of Lamin C and subnuclear localization of Ser22-phosphorylated Lamin C did not appear to change. Thus, it remains unclear how Ser22-phosphorylated Lamin C is misdirected in progeria. Given the observation that progerin directly interacts with Lamin C [144], one possibility is that the progerin–Lamin C interaction alters the binding specificity of Lamin C. Several chemical compounds known to inhibit the interaction between Lamin A/C and progerin [144] may be useful for examining this possibility.

## Laminopathy mutations that affect lamin phosphorylation

There are multiple ways in which laminopathy mutations could affect nuclear lamin phosphorylation. First, laminopathy mutations could lead to nonsynonymous substitutions of the residues subject to phosphorylation. Such pathogenic substitutions in Lamin A/C include Thr10Ile associated with lipodystrophy [145], Ser22Leu associated with dilated cardiomyopathy [146], Ser27Ile associated with limb-girdle muscular dystrophy with dilated cardiomyopathy [147], Ser143Phe associated with congenital muscular dystrophy [148], and Thr528Lys associated with Emery-Dreifuss muscular dystrophy [59,149] (Supplementary Table 1). Whether these mutations affect assembly or localization of Lamin A/C has not to date been characterized. Pathogenic missense substitutions in Lamin A/C are apparently not over-represented among phosphorylation sites, potentially due to the critical role of Lamin A/C phosphorylation during mitosis (pathogenic substitutions overlap 22% of phosphorylation sites vs. 42% of non-phosphorylation sites. Pathogenic site data from http://www.umd.be/LMNA/). Second, pathogenic mutations may alter kinase-recognition motifs surrounding phosphorylation sites. While this scenario has not been explored in detail, Lin et al. predicted that pathogenic *LMNA* mutations decrease Lamin A/C phosphorylation overall in an *in silico* analysis [150]. Third, pathogenic mutations may alter the accessibility of kinases or phosphatases to target residues through protein conformation changes. Mitsuhashi et al. reported that Ser458, a site within the Ig fold domain, is phosphorylated in the muscle tissues of *LMNA*-related muscular-dystrophy patients only when mutations are located within the Ig fold [90]. In contrast, Ser458 was not phosphorylated in neuromuscular disorders unrelated to *LMNA* mutations or cells expressing mutant Lamin A that causes non-myopathic laminopathies [90]. The authors found that the kinase Akt1 phosphorylates Ser458 and speculated that myopathy-causing *LMNA* mutations render Ser458 accessible to Akt1 through a conformational change of the Ig fold domain [90]. However, Ser458 phosphorylation has later been reported in *LMNA*-wild-type HeLa cells [27]. Finally, there are reports that laminopathy mutations are associated with changes in Lamin A/C phosphorylation at undefined residues. Cenni et al. reported strong reduction of overall Lamin A/C phosphorylation in myoblasts and myotubes derived from various muscular dystrophy patients with *LMNA* gene mutations [89]. Lin et al. reported that R60G substitution, which causes dilated cardiomyopathy, renders this mutant protein more resistant to phosphorylation by p38 MAPK at undefined sites [150]. These studies highlight multiple ways in which pathogenic *LMNA* mutations could affect Lamin A/C phosphorylation. Overall, however, only a small number of studies have investigated the impact of the laminopathy mutations on lamin phosphorylation. Therefore, an unbiased survey of the phosphorylation state of Lamin A/C in tissues affected in laminopathies is warranted.

## Conclusion & outlook

Our review catalogs an extensive repertoire of phosphorylation sites in nuclear lamins as well as kinases and phosphatases that regulate lamin phosphorylation (Supplementary Table 1). We recognize a wide variety of biological contexts that promote lamin phosphorylation during interphase Figure 2. Instead of phosphorylated lamins solely existing as byproducts of lamin disassembly during mitosis, an emerging hypothesis posits that phosphorylated lamins have specific functions in the nuclear interior in interphase cells. One example of such a function is the binding of Ser22-phosphorylated lamin C to the genomic regions characteristic of active enhancers near transcriptionally active genes [28]. Recent studies highlight multiple ways in which laminopathy mutations are predicted to affect lamin phosphorylation and functions of phosphorylated lamins. Thus, lamin phosphorylation presents a new avenue to investigate lamin functions in the cell and the molecular basis for the pathogenesis of laminopathies.

Our review also clarifies a number of important questions that have been left unanswered. These questions concern the biological contexts in which nuclear lamin phosphorylation is regulated by specific kinases and phosphatases; the subcellular locations at which these kinases and phosphatases operate on lamins; the functions of phosphorylated nuclear lamins at chromatin and in other cellular processes; and the mechanisms by which laminopathy mutations affect lamin phosphorylation and the functions of phosphorylated lamins. Addressing these questions will require the development of new tools and techniques, such as phosphorylation-specific antibodies, manipulation of phosphorylation states *in vivo*, and a proteome-wide survey of lamin phosphorylation in normal and disease tissues. Finally, it should be noted that other post-translational modifications, such as sumoylation, acetylation, and ADP-ribosylation, have been reported for lamins but not studied as extensively as phosphorylation [41,151]. We anticipate a new endeavor to characterize functions and regulatory mechanisms of various post-translational modifications of nuclear lamins in the coming years.

## Supplementary Material

Supplemental Material
