# Supplementary material for: Nuclear lamin phosphorylation: an emerging role in gene regulation and pathogenesis of laminopathies
Source: Nucleus. 2020 Oct 25;11(1):299–314. doi: 10.1080/19491034.2020.1832734 (PMC7588210; doi:10.1080/19491034.2020.1832734)
Supplement: Supplemental Material [file KNCL_A_1832734_SM8315.docx]

# **SUPPLEMENTARY TABLE 1**

| **Phosphorylation site in Lamin A/C** | | | | | | | |
| --- | --- | --- | --- | --- | --- | --- | --- |
| **Amino acid**  **(P02545)** | **Lamin A/C^a)^** | **Interphase/**  **mitosis** | **Kinase** | **Phosphatase** | **Disease mutation^b)^** | **Interphase subnuclear localization** | **Additional note** |
| **T3** | AC | I [^1^](https://paperpile.com/c/dg0lOH/wgbwn) & M [^2^](https://paperpile.com/c/dg0lOH/lYxnO) |  | PP2A [^3^](https://paperpile.com/c/dg0lOH/gy0YM) |  |  |  |
| **S5** | AC | I [^1,4^](https://paperpile.com/c/dg0lOH/wgbwn+MNFot) | PKC [^4^](https://paperpile.com/c/dg0lOH/MNFot) |  |  |  |  |
| **T10** | AC | I [^1^](https://paperpile.com/c/dg0lOH/wgbwn) & M [^4^](https://paperpile.com/c/dg0lOH/MNFot) | Akt-RSK-p70S6 Kinase network [^5^](https://paperpile.com/c/dg0lOH/C31hW), PKA [^4^](https://paperpile.com/c/dg0lOH/MNFot) |  | APS (T10I) |  |  |
| **S12** | AC | I [^1^](https://paperpile.com/c/dg0lOH/wgbwn) & M [^2^](https://paperpile.com/c/dg0lOH/lYxnO) | Akt-RSK-p70S6 Kinase network [^5^](https://paperpile.com/c/dg0lOH/C31hW) |  |  | Phospho-mimetic or phospho-deficient substitution does not affect localization [^1^](https://paperpile.com/c/dg0lOH/wgbwn) |  |
| **S17** | AC |  |  |  |  |  |  |
| **S18** | AC | I [^1^](https://paperpile.com/c/dg0lOH/wgbwn) & M [^2^](https://paperpile.com/c/dg0lOH/lYxnO) |  |  |  |  |  |
| **T19** | AC | I [^1^](https://paperpile.com/c/dg0lOH/wgbwn)& M [^2,4^](https://paperpile.com/c/dg0lOH/MNFot+lYxnO) | CDK1 [^4^](https://paperpile.com/c/dg0lOH/MNFot) | PP2A [^3^](https://paperpile.com/c/dg0lOH/gy0YM) |  |  |  |
| **S22** | AC | I [^1^](https://paperpile.com/c/dg0lOH/wgbwn) & M [^2,6^](https://paperpile.com/c/dg0lOH/lYxnO+ZWULV) | CDK1 [^7,8^](https://paperpile.com/c/dg0lOH/pWnkC+H3VOV), CDK5 [^9^](https://paperpile.com/c/dg0lOH/P7Ol5), ERK2 [^10^](https://paperpile.com/c/dg0lOH/FVgzP), BGLF4 [^11^](https://paperpile.com/c/dg0lOH/HRbRY) | PP2A [^3^](https://paperpile.com/c/dg0lOH/gy0YM) | CMD1A (S22L) | S22-phosphorylated Lamin A/C localizes in the nuclear interior [^1,12^](https://paperpile.com/c/dg0lOH/wgbwn+Y18qR) |  |
| **T24** | AC | M [^2^](https://paperpile.com/c/dg0lOH/lYxnO) |  |  |  |  |  |
| **T27** | AC |  |  |  | EDMD (T27I) |  |  |
| **S51** | AC |  |  |  |  |  |  |
| **T54** | AC |  |  |  |  |  |  |
| **T64** | AC |  |  |  |  |  |  |
| **S66** | AC |  |  |  |  |  |  |
| **S71** | AC |  |  |  |  |  |  |
| **Y81** | AC |  |  |  | CMD1A (Y81F) |  |  |
| **S94** | AC |  |  |  |  |  |  |
| **S107** | AC |  |  |  |  |  |  |
| **S143** | AC |  |  |  | APS (S143F);  CMD1A (S143P) |  |  |
| **S153** | AC |  |  |  |  |  |  |
| **T157** | AC |  |  |  | EDMD + neuropathies (T157T; 471G>A possible splicing activation) |  |  |
| **T199** | AC |  | PKC [^4^](https://paperpile.com/c/dg0lOH/MNFot) |  |  |  |  |
| **S212** | AC |  |  |  |  |  |  |
| **T218** | AC |  |  |  |  |  |  |
| **S277** | AC |  |  |  | EDMD (S277P) |  |  |
| **S282** | AC |  |  | PP2A [^3^](https://paperpile.com/c/dg0lOH/gy0YM) |  |  |  |
| **S295** | AC |  |  |  | EDMD (S295P) |  |  |
| **S301** | AC |  | Akt [^13^](https://paperpile.com/c/dg0lOH/TCaUo);  Akt-RSK-p70S6 Kinase network [^5^](https://paperpile.com/c/dg0lOH/C31hW) |  |  |  |  |
| **S303** | AC |  | Akt-RSK-p70S6 Kinase network [^5^](https://paperpile.com/c/dg0lOH/C31hW) |  | EDMD (S303P) |  |  |
| **S307** | AC |  | Chk1 [^14^](https://paperpile.com/c/dg0lOH/nHiKa) |  |  |  |  |
| **S326** | AC |  |  |  | CMD1A (S326T);  EDMD (S326T);  CMD1A (S326L) |  |  |
| **S390** | AC | I [^1,4^](https://paperpile.com/c/dg0lOH/wgbwn+MNFot) | CDK1 [^4^](https://paperpile.com/c/dg0lOH/MNFot),  Akt-RSK-p70S6 Kinase network [^5^](https://paperpile.com/c/dg0lOH/C31hW), BGLF4 [^11^](https://paperpile.com/c/dg0lOH/HRbRY) | PP2A [^3^](https://paperpile.com/c/dg0lOH/gy0YM) |  | Phospho-deficient substitution reduces nuclear Interior localization [^1^](https://paperpile.com/c/dg0lOH/wgbwn) |  |
| **S392** | AC | I [^1^](https://paperpile.com/c/dg0lOH/wgbwn) & M [^4^](https://paperpile.com/c/dg0lOH/MNFot) | CDK1[^4^](https://paperpile.com/c/dg0lOH/MNFot), CDK5 [^9^](https://paperpile.com/c/dg0lOH/P7Ol5),  Akt-RSK-p70S6 Kinase network [^5^](https://paperpile.com/c/dg0lOH/C31hW),  BGLF4 [^11^](https://paperpile.com/c/dg0lOH/HRbRY) | PP2A [^3^](https://paperpile.com/c/dg0lOH/gy0YM) |  | Phospho-mimetic substitution increases nuclear Interior localization [^1^](https://paperpile.com/c/dg0lOH/wgbwn) |  |
| **T394** | AC | M [^2^](https://paperpile.com/c/dg0lOH/lYxnO) |  |  |  |  |  |
| **S395** | AC | I [^4^](https://paperpile.com/c/dg0lOH/MNFot) & M [^2,4^](https://paperpile.com/c/dg0lOH/MNFot+lYxnO) | PKC [^4^](https://paperpile.com/c/dg0lOH/MNFot) |  | Metabolic Disorder (S395L) |  |  |
| **S398** | AC |  | Akt-RSK-p70S6 Kinase network [^5^](https://paperpile.com/c/dg0lOH/C31hW) |  |  |  |  |
| **S403** | AC | I [^1^](https://paperpile.com/c/dg0lOH/wgbwn) & M [^2^](https://paperpile.com/c/dg0lOH/lYxnO) | Akt-RSK-p70S6 Kinase network [^5^](https://paperpile.com/c/dg0lOH/C31hW) |  |  |  | Phospho-deficient S403A substitution reduces nuclear import [^15^](https://paperpile.com/c/dg0lOH/6hA4w) |
| **S404** | AC | I [^1^](https://paperpile.com/c/dg0lOH/wgbwn) & M [^2^](https://paperpile.com/c/dg0lOH/lYxnO) | Akt [^13^](https://paperpile.com/c/dg0lOH/TCaUo), Akt-RSK-p70S6 Kinase network [^5^](https://paperpile.com/c/dg0lOH/C31hW) |  |  | Phospho-mimetic substitutions in both S404 and S407 increase nuclear Interior localization  [^1^](https://paperpile.com/c/dg0lOH/wgbwn) | Phospho-deficient S404A substitution reduces nuclear import (additively reduced in double S403A/S404A mutation) [^15^](https://paperpile.com/c/dg0lOH/6hA4w); S404A forms blebs and honeycomb structures in the nucleus [^13^](https://paperpile.com/c/dg0lOH/TCaUo) |
| **S406** | AC | I [^1^](https://paperpile.com/c/dg0lOH/wgbwn) & M [^2^](https://paperpile.com/c/dg0lOH/lYxnO) | Akt-RSK-p70S6 Kinase network [^5^](https://paperpile.com/c/dg0lOH/C31hW) |  |  |  |  |
| **S407** | AC | I [^1,4^](https://paperpile.com/c/dg0lOH/MNFot+wgbwn)& M [^2,4^](https://paperpile.com/c/dg0lOH/MNFot+lYxnO) | Akt-RSK-p70S6 Kinase network [^5^](https://paperpile.com/c/dg0lOH/C31hW) |  |  | Phospho-mimetic substitutions in both S404 and S407 increase nuclear Interior localization [^1^](https://paperpile.com/c/dg0lOH/wgbwn) |  |
| **T409** | AC | I [^4^](https://paperpile.com/c/dg0lOH/MNFot) & M [^2,4^](https://paperpile.com/c/dg0lOH/MNFot+lYxnO) | Akt-RSK-p70S6 Kinase network [^5^](https://paperpile.com/c/dg0lOH/C31hW) |  |  |  |  |
| **S414** | AC | M [^2^](https://paperpile.com/c/dg0lOH/lYxnO) | Akt-RSK-p70S6 Kinase network [^5^](https://paperpile.com/c/dg0lOH/C31hW) |  |  |  |  |
| **T416** | AC | I [^1,4^](https://paperpile.com/c/dg0lOH/MNFot+wgbwn) & M [^2,4^](https://paperpile.com/c/dg0lOH/MNFot+lYxnO) | PKC [^4^](https://paperpile.com/c/dg0lOH/MNFot), Akt-RSK-p70S6 Kinase network [^5^](https://paperpile.com/c/dg0lOH/C31hW) |  |  |  |  |
| **S423** | AC | I [^1^](https://paperpile.com/c/dg0lOH/wgbwn) |  |  |  | Phospho-mimetic or phospho-deficient substitution does not affect localization [^1^](https://paperpile.com/c/dg0lOH/wgbwn) |  |
| **T424** | AC |  |  |  |  |  |  |
| **S426** | AC | I [^1^](https://paperpile.com/c/dg0lOH/wgbwn) |  |  |  |  |  |
| **S428** | AC |  |  |  |  |  |  |
| **S429** | AC |  |  |  |  |  |  |
| **S431** | AC | M [^2^](https://paperpile.com/c/dg0lOH/lYxnO) |  |  |  |  |  |
| **T436** | AC |  |  |  |  |  |  |
| **S437** | AC |  |  |  |  |  |  |
| **S458** | AC | I [^1^](https://paperpile.com/c/dg0lOH/wgbwn) | Akt [^16^](https://paperpile.com/c/dg0lOH/NRM8f),  Akt-RSK-p70S6 Kinase network [^5^](https://paperpile.com/c/dg0lOH/C31hW) |  |  |  |  |
| **S463** | AC |  |  |  |  |  |  |
| **T480** | AC | I [^4^](https://paperpile.com/c/dg0lOH/MNFot) | PKC [^4^](https://paperpile.com/c/dg0lOH/MNFot), Akt-RSK-S6 Kinase network [^5^](https://paperpile.com/c/dg0lOH/C31hW) |  |  |  |  |
| **T505** | AC | M [^2^](https://paperpile.com/c/dg0lOH/lYxnO) |  |  |  |  |  |
| **S507** | AC | M [^4^](https://paperpile.com/c/dg0lOH/MNFot) |  |  |  |  |  |
| **S525** | AC | I [^4^](https://paperpile.com/c/dg0lOH/MNFot) | PKC [^4^](https://paperpile.com/c/dg0lOH/MNFot) |  |  | Phospho-deficient substitutions exhibited increased and abnormal aggregation in the nuclear interior, as well as a discontinuous distribution at the nuclear lamina [^17^](https://paperpile.com/c/dg0lOH/LA3Rp) |  |
| **T528** | AC |  |  |  | CMD1A (T528L); EDMD (T528L);  EDMD (T528R); L-CMD (T528R); FPLD2 (T528M); APS or HGPS (T528M); CMD1A (T528M) |  |  |
| **S533** | AC |  |  |  |  |  |  |
| **T534** | AC |  |  |  | EDMD (T534S) |  |  |
| **S546** | AC |  | Akt-RSK-p70S6 Kinase network [^5^](https://paperpile.com/c/dg0lOH/C31hW) |  |  |  |  |
| **T548** | AC |  | Akt-RSK-p70S6 Kinase network [^5^](https://paperpile.com/c/dg0lOH/C31hW) |  |  |  |  |
| **S568** | C |  |  |  |  |  |  |
| **S570** | C | I [^1,4^](https://paperpile.com/c/dg0lOH/MNFot+wgbwn) & M [^4^](https://paperpile.com/c/dg0lOH/MNFot) | PKC [^4^](https://paperpile.com/c/dg0lOH/MNFot) |  |  |  | S572 in mouse |
| **S571** | A |  |  |  | CMT2B1 + EDMD + FPLD2 (S571C); CMT2B1 + FPLD2 (S571C); CMD1A (S571R)) |  |  |
| **S572** | A |  |  |  | LVNC (S572C);  CMD1A (S572C) |  |  |
| **S573** | A |  |  |  | HCM + Metabolic Syndrome (S573L);  CMD1A (S573L);  APS (S573L);  FPLD2 (S573L);  EDMD (S573L); |  |  |
| **S576** | A |  |  |  |  |  |  |
| **E578** | A |  |  |  | APS(E578V) |  |  |
| **S583** | A |  |  |  | FPLD2 (S583L) |  |  |
| **T585** | A |  |  |  |  |  |  |
| **T590** | A |  |  |  |  |  |  |
| **S599** | A |  |  |  |  |  |  |
| **S601** | A |  |  |  |  |  |  |
| **S603** | A |  |  |  |  |  |  |
| **S612** | A |  |  |  |  |  |  |
| **S613** | A |  |  |  |  |  |  |
| **S615** | A | M [^2^](https://paperpile.com/c/dg0lOH/lYxnO) |  |  |  |  |  |
| **S616** | A | M [^2^](https://paperpile.com/c/dg0lOH/lYxnO) |  |  |  |  |  |
| **S618** | A |  |  |  |  |  |  |
| **S619** | A | M [^2^](https://paperpile.com/c/dg0lOH/lYxnO) |  |  |  |  |  |
| **T621** | A |  |  |  |  |  |  |
| **T623** | A |  |  |  | HGPS (T623S);  Note demonstrated splicing activation. |  |  |
| **S625** | A |  | PKC [^4^](https://paperpile.com/c/dg0lOH/MNFot) |  |  |  |  |
| **S628** | A | I [^1^](https://paperpile.com/c/dg0lOH/wgbwn) & M [^2^](https://paperpile.com/c/dg0lOH/lYxnO) |  | PP2A [^3^](https://paperpile.com/c/dg0lOH/gy0YM) |  |  | Phospho-mimetic substitution increases cytoplasmic localization when combined with phospho-mimetic substitutions in S22 and S392 [^1^](https://paperpile.com/c/dg0lOH/wgbwn) |
| **S632** | A | M [^2^](https://paperpile.com/c/dg0lOH/lYxnO) |  | PP2A [^3^](https://paperpile.com/c/dg0lOH/gy0YM) |  |  |  |
| **S636** | A | I [^1^](https://paperpile.com/c/dg0lOH/wgbwn) & M [^2^](https://paperpile.com/c/dg0lOH/lYxnO) |  | PP2A [^3^](https://paperpile.com/c/dg0lOH/gy0YM) |  |  |  |
| **S645** | A |  |  |  |  |  |  |
| **T643** | A |  |  |  | CMT2B1 (autosomal recessive) (Thr643N) |  |  |
| **S651** | A |  |  |  |  |  | S652 in mouse |
| **S652** | A | I [^1^](https://paperpile.com/c/dg0lOH/wgbwn) & M [^2^](https://paperpile.com/c/dg0lOH/lYxnO) | PKC [^4^](https://paperpile.com/c/dg0lOH/MNFot), BGLF4 [^11^](https://paperpile.com/c/dg0lOH/HRbRY) | PP2A [^3^](https://paperpile.com/c/dg0lOH/gy0YM) |  |  |  |
| **S657** | A |  | BGLF4 [^11^](https://paperpile.com/c/dg0lOH/HRbRY) |  |  |  |  |

##

a) A, Lamin A; C, Lamin C; AC, Lamin A and C

b) Disease mutations are obtained from [http://www.umd.be/LMNA](http://www.umd.be/LMNA/) (version January 31, 2020). Abbreviations for phenotypic classifications are: APS, atypical progeroid syndrome; CMD1A, dilated cardiomyopathy 1A; CMT2B1, Charcot-Marie-Tooth neuropathy, type 2B1; EDMD, Emery-Dreifuss muscular dystrophy 2 or 3; FPLD2, Familial partial lipodystrophy type 2; HGPS, Hutchinson-Gilford progeria syndrome; L-CMD, *LMNA*-related congenital muscular dystrophy; HCM, hypertrophic cardiomyopathy, LVNC, left ventricular non-compaction cardiomyopathy

1. [Kochin V, Shimi T, Torvaldson E, Adam SA, Goldman A, Pack C-G, Melo-Cardenas J, Imanishi SY, Goldman RD, Eriksson JE. Interphase phosphorylation of lamin A. J Cell Sci [Internet] 2014; 127:2683–96. Available from:](http://paperpile.com/b/dg0lOH/wgbwn) <http://dx.doi.org/10.1242/jcs.141820>

2. [Olsen JV, Vermeulen M, Santamaria A, Kumar C, Miller ML, Jensen LJ, Gnad F, Cox J, Jensen TS, Nigg EA, et al. Quantitative phosphoproteomics reveals widespread full phosphorylation site occupancy during mitosis. Sci Signal [Internet] 2010; 3:ra3. Available from:](http://paperpile.com/b/dg0lOH/lYxnO) <http://dx.doi.org/10.1126/scisignal.2000475>

3. [Kauko O, Imanishi SY, Kulesskiy E, Yetukuri L, Laajala TD, Sharma M, Pavic K, Aakula A, Rupp C, Jumppanen M, et al. Phosphoproteome and drug-response effects mediated by the three protein phosphatase 2A inhibitor proteins CIP2A, SET, and PME-1. J Biol Chem [Internet] 2020; 295:4194–211. Available from:](http://paperpile.com/b/dg0lOH/gy0YM) <http://dx.doi.org/10.1074/jbc.RA119.011265>

4. [Eggert M, Radomski N, Linder D, Tripier D, Traub P, Jost E. Identification of novel phosphorylation sites in murine A-type lamins. Eur J Biochem [Internet] 1993; 213:659–71. Available from:](http://paperpile.com/b/dg0lOH/MNFot) <http://dx.doi.org/10.1111/j.1432-1033.1993.tb17806.x>

5. [Moritz A, Li Y, Guo A, Villén J, Wang Y, MacNeill J, Kornhauser J, Sprott K, Zhou J, Possemato A, et al. Akt-RSK-S6 kinase signaling networks activated by oncogenic receptor tyrosine kinases. Sci Signal [Internet] 2010; 3:ra64. Available from:](http://paperpile.com/b/dg0lOH/C31hW) <http://dx.doi.org/10.1126/scisignal.2000998>

6. [Ward GE, Kirschner MW. Identification of cell cycle-regulated phosphorylation sites on nuclear lamin C. Cell [Internet] 1990; 61:561–77. Available from:](http://paperpile.com/b/dg0lOH/ZWULV) <https://www.ncbi.nlm.nih.gov/pubmed/2188730>

7. [Peter M, Nakagawa J, Dorée M, Labbé JC, Nigg EA. In vitro disassembly of the nuclear lamina and M phase-specific phosphorylation of lamins by cdc2 kinase. Cell [Internet] 1990; 61:591–602. Available from:](http://paperpile.com/b/dg0lOH/pWnkC) <https://www.ncbi.nlm.nih.gov/pubmed/2188731>

8. [Heald R, McKeon F. Mutations of phosphorylation sites in lamin A that prevent nuclear lamina disassembly in mitosis. Cell [Internet] 1990; 61:579–89. Available from:](http://paperpile.com/b/dg0lOH/H3VOV) <https://www.ncbi.nlm.nih.gov/pubmed/2344612>

9. [Chang K-H, Multani PS, Sun K-H, Vincent F, de Pablo Y, Ghosh S, Gupta R, Lee H-P, Lee H-G, Smith MA, et al. Nuclear envelope dispersion triggered by deregulated Cdk5 precedes neuronal death. Mol Biol Cell [Internet] 2011; 22:1452–62. Available from:](http://paperpile.com/b/dg0lOH/P7Ol5) <http://dx.doi.org/10.1091/mbc.E10-07-0654>

10. [Carlson SM, Chouinard CR, Labadorf A, Lam CJ, Schmelzle K, Fraenkel E, White FM. Large-scale discovery of ERK2 substrates identifies ERK-mediated transcriptional regulation by ETV3. Sci Signal [Internet] 2011; 4:rs11. Available from:](http://paperpile.com/b/dg0lOH/FVgzP) <http://dx.doi.org/10.1126/scisignal.2002010>

11. [Lee C-P, Huang Y-H, Lin S-F, Chang Y, Chang Y-H, Takada K, Chen M-R. Epstein-Barr virus BGLF4 kinase induces disassembly of the nuclear lamina to facilitate virion production. J Virol [Internet] 2008; 82:11913–26. Available from:](http://paperpile.com/b/dg0lOH/HRbRY) <http://dx.doi.org/10.1128/JVI.01100-08>

12. [Ikegami K, Secchia S, Almakki O, Lieb JD, Moskowitz IP. Phosphorylated Lamin A/C in the Nuclear Interior Binds Active Enhancers Associated with Abnormal Transcription in Progeria. Dev Cell [Internet] 2020; 52:699–713.e11. Available from:](http://paperpile.com/b/dg0lOH/Y18qR) <http://dx.doi.org/10.1016/j.devcel.2020.02.011>

13. [Cenni V, Bertacchini J, Beretti F, Lattanzi G, Bavelloni A, Riccio M, Ruzzene M, Marin O, Arrigoni G, Parnaik V, et al. Lamin A Ser404 is a nuclear target of Akt phosphorylation in C2C12 cells. J Proteome Res [Internet] 2008; 7:4727–35. Available from:](http://paperpile.com/b/dg0lOH/TCaUo) <http://dx.doi.org/10.1021/pr800262g>

14. [Blasius M, Forment JV, Thakkar N, Wagner SA, Choudhary C, Jackson SP. A phospho-proteomic screen identifies substrates of the checkpoint kinase Chk1. Genome Biol [Internet] 2011; 12:R78. Available from:](http://paperpile.com/b/dg0lOH/nHiKa) <http://dx.doi.org/10.1186/gb-2011-12-8-r78>

15. [Leukel M, Jost E. Two conserved serines in the nuclear localization signal flanking region are involved in the nuclear targeting of human lamin A. Eur J Cell Biol [Internet] 1995; 68:133–42. Available from:](http://paperpile.com/b/dg0lOH/6hA4w) <https://www.ncbi.nlm.nih.gov/pubmed/8575460>

16. [Mitsuhashi H, Hayashi YK, Matsuda C, Noguchi S, Wakatsuki S, Araki T, Nishino I. Specific phosphorylation of Ser458 of A-type lamins in LMNA-associated myopathy patients. J Cell Sci [Internet] 2010; 123:3893–900. Available from:](http://paperpile.com/b/dg0lOH/NRM8f) <http://dx.doi.org/10.1242/jcs.072157>

17. [Haas M, Jost E. Functional analysis of phosphorylation sites in human lamin A controlling lamin disassembly, nuclear transport and assembly. Eur J Cell Biol [Internet] 1993; 62:237–47. Available from:](http://paperpile.com/b/dg0lOH/LA3Rp) <https://www.ncbi.nlm.nih.gov/pubmed/7925482>
